# Tailoring the physical properties of Ni-based single-phase equiatomic alloys by modifying the chemical complexity

**DOI:** 10.1038/srep20159

**Published:** 2016-02-01

**Authors:** K. Jin, B. C. Sales, G. M. Stocks, G. D. Samolyuk, M. Daene, W. J. Weber, Y. Zhang, H. Bei

**Affiliations:** 1 Materials Science & Technology Division, Oak Ridge National Laboratory, Oak Ridge, TN 37831, USA; 2 Department of Materials Science & Engineering, University of Tennessee, Knoxville, TN 37996, USA; 3 Physical and Life Sciences, Lawrence Livermore National Laboratory, Livermore, CA 94551, United State

## Abstract

Equiatomic alloys (e.g. high entropy alloys) have recently attracted considerable interest due to their exceptional properties, which might be closely related to their extreme disorder induced by the chemical complexity. In order to understand the effects of chemical complexity on their fundamental physical properties, a family of (eight) Ni-based, face-center-cubic (FCC), equiatomic alloys, extending from elemental Ni to quinary high entropy alloys, has been synthesized, and their electrical, thermal, and magnetic properties are systematically investigated in the range of 4–300 K by combining experiments with *ab initio* Korring-Kohn-Rostoker coherent-potential-approximation (KKR-CPA) calculations. The scattering of electrons is significantly increased due to the chemical (especially magnetic) disorder. It has weak correlation with the number of elements but strongly depends on the type of elements. Thermal conductivities of the alloys are largely lower than pure metals, primarily because the high electrical resistivity suppresses the electronic thermal conductivity. The temperature dependence of the electrical and thermal transport properties is further discussed, and the magnetization of five alloys containing three or more elements is measured in magnetic fields up to 4 T.

Recently, a new family of compositionally complex (containing 4, 5, or more elements) but structurally simple (e.g. face center cubic - FCC structured) alloys, such as high-entropy alloys (HEA), has been successfully fabricated, in which the atomic fraction of each component is equal or near-equal^1^,^2^,^3^,^4^,^5^,^6^,^7^. Therefore, the knowledge obtained from traditional solid solution alloys with distinguishable “solvent” and “solute” species may not be suitable to describe those alloys, and their mechanical and physical properties may be unique with potential practical applications. For example, in contrast to most traditional materials in which an inverse temperature-dependence of strength and ductility is usually observed, recent mechanical testing has shown that the FCC NiCoFeCrMn HEA shows simultaneous increases in both strength and ductility with decreasing test temperature (e.g. from 293 to 77 K). This alloy also has excellent fracture toughness at liquid nitrogen temperature, as high as 200 MPa m^1/2^, which is comparable to the very best cryogenic steels^7^,^8^. In addition to the extraordinary mechanical properties, these novel materials have also been proposed for other applications such as soft ferromagnetic materials (SFM)^9^ and radiation resistant nuclear materials^10^. In order to investigate the potential of such applications, knowledge of their fundamental physical properties is highly desired. For example, thermal conductivity is a crucial parameter needed in the simulation of defect evolution under irradiation (or energy deposition) processes, and believed to be related to the chemical complexity of the alloys^11^.

From a scientific perspective, electrical and thermal transport properties along with the magnetization of substitutionally disordered crystalline alloys, especially the ones with multiple concentrated magnetic metals, are of fundamental interest. For example, the residual electrical resistivity of these alloys is usually 1–2 orders greater than the pure metal and its dilute alloys^12^,^13^,^14^, but whether potential scattering from chemical disorder, scattering from lattice disorder, or both, are the dominant scattering mechanisms is not fully understood. The temperature dependence of electrical resistivity, especially at low temperature (*T*), is more complicated because of the mixture of electron-phonon, electron-electron, and electron-magnon interactions^12^,^15^,^16^. In addition, the magnetic phase of such alloys could be complex and sensitive to the change in compositions. For example, with varying Fe concentrations, Fe_x_-Ni_80-x_-Cr_20_ alloys show different magnetic phases of ferromagnetic, antiferromagnetic, paramagnetic and spin glass^17^.

In the 1970’s, experiments on concentrated alloys largely focused on binary systems^15^,^16^. More recently, ternary systems were investigated, particularly the Ni-Fe-Cr system because Ni, Fe, Cr are the major elements in many important commercial alloys, e.g. stainless steels^12^,^18^. The physical properties of a few high entropy alloys have been studied recently, but these studies have either focused on room or higher temperatures (NiCoFeCrCu and NiCoFeCrPd)^9^,^19^,^20^, or on one alloy system where the concentration of one element is varied (NiCoFeCrAl_x_)^13^. Moreover, NiCoFeCrCu and NiCoFeCrAl alloys normally do not have simple microstructures; rather they consist of multiple phases in the materials. Theoretically, *ab initio* approaches applied in concentrated random solid solution alloys have been developed in the 1980’s, in which the coherent-potential approximation (CPA), an effective medium theory, is used to describe the effects of compositional disorder on the underlying electronic structure. Early applications to binary systems, using for example the (Korringa-Kohn-Rostoker) KKR-CPA, have shown good agreement with experimental results of the electronic structures (e.g. by X-ray spectroscopy)^21^,^22^,^23^. Furthermore, calculated transport properties compared well with experimental values^14^,^21^,^24^. In recent years, the CPA calculations have been successfully expanded to the applications in high entropy alloys, with majority of them focused on the structural, mechanical, defect, and magnetic properties^25^,^26^,^27^,^28^,^29^,^30^.

Extreme chemical complexity is one of the key features of these concentrated (particularly equiatomic) high entropy alloys. Their unique properties have been generally discussed from perspectives of alloy complexity, where the controlling factors are the number and the type of elements comprising the alloys. For example, the diffusion activation energy has been reported to be positively related to the number of elements in the matrix^31^, while the hardening effect of HEAs (e.g. NiCoFeCrMn) has been considered to result from size/modulus mismatch between the alloying elements; in this case, certain alloying species (e.g. Cr) are more critical than the number of elements^32^,^33^. In the case of physical properties, however, the general picture of the effects of chemical complexity on magnetism and electrical and thermal transport, especially at low temperatures, is still unclear in this new family of alloys. Moreover, considering the fact that CPA does not include the displacement fluctuation, whether or not it can capture the major feature of the transport properties in these compositionally complex alloys remains to be explored. Here a series of Ni-based equiatomic FCC alloys are selected for investigation, including NiCoFeCrPd, NiCoFeCrMn, NiCoFeCr, NiCoCr, NiCoFe, NiFe, and NiCo, as well as elemental Ni. These alloys are selected in part because recent experiments confirmed that these equiatomic alloys form a single-phase solid-solution with the simple FCC crystal structure^32^,^34^. These alloys thus provide an ideal system to systematically study how the type and number of elements affect the transport properties of equiatomic alloys. Electrical and thermal transport data are reported for all of the alloys for temperatures between 4 and 300 K, and *ab initio* KKR- CPA calculations are performed to investigate the origin of high residual resistivity (ρ_R_, resistivity at 0 K). The Wiedemann-Franz relationship is used to estimate the phonon and electron contributions to the measured total thermal conductivity. In addition, magnetization data are reported for the five alloys with three or more elements from 4 to 300 K using applied fields between 0 and 4 Tesla.

## Experimental Results

### Electrical resistivity

**Figure 1:**
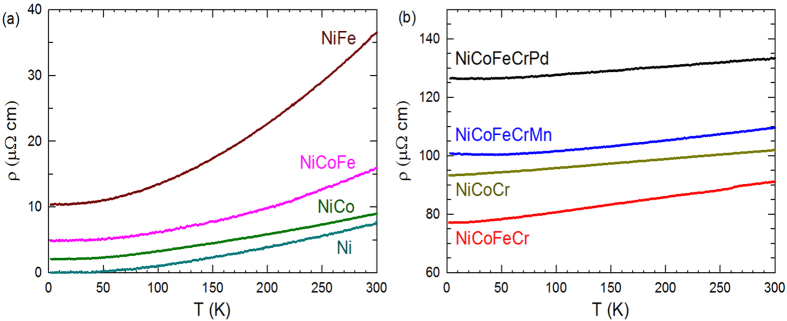
Electrical resistivity in Ni-based FCC equiatomic alloys. Electrical resistivity from 4 to 300 K of (**a**) Ni, NiCo, NiFe and NiCoFe, and (**b**) NiCoCr, NiCoFeCr, NiCoFeCrMn and NiCoFeCrPd. The materials in (**a**) have significantly lower resistivity than those in (**b**).

The electrical resistivity, ρ(*T*), in the temperature range of 4–300 K is shown in Fig. 1 for the eight measured materials. The measured values of ρ(*T*) for Ni, NiCo and NiFe are consistent with literature results^16^,^35^. The measured resistivities for Ni, NiCo, NiCoFe and NiFe in Fig. 1(a) are about one order of magnitude smaller than those measured for the other four alloys containing chromium, NiCoFeCr, NiCoCr, NiCoFeCrMn and NiCoFeCrPd, as shown in Fig. 1(b).

The temperature dependence of the electrical resistivity varies in different temperature regimes. At sufficiently high temperatures, the Bloch-Grüneissen theory predicts a linear temperature dependence of resistivity due to the scattering from phonons^16^. As the temperature is decreased, a *T*^2^ dependence becomes significant in many transition metals or concentrated transition-metal alloys^12^,^18^. For ferromagnetic metals, this can be attributed to spin-wave scattering, and the theoretical estimations agree well with experiments in the cases of elemental Ni, Co and Fe^36^,^37^,^38^. The *T*^2^ contribution could also arise from nonmagnetic origins in the case of strong electron-electron scattering^16^,^39^. If Matthiessen’s rule holds, in the high temperature regime (>100 K), the measured electrical resistivity can be described by,


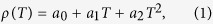


where *a*_0_*, a*_1_, and *a*_2_ are fitting parameters determined from a least square fit to the data. These parameters are listed in Table 1, where the resistivity values at 300 K are also included. Note that *a*_0_ here is not the residual resistivity. Negligible values of parameter *a*_2_ for NiCoCr, NiCoFeCr and NiCoFeCrPd indicates good linearity in this temperature range, where the *T*^2^ term is not significant.

**Figure 2:**
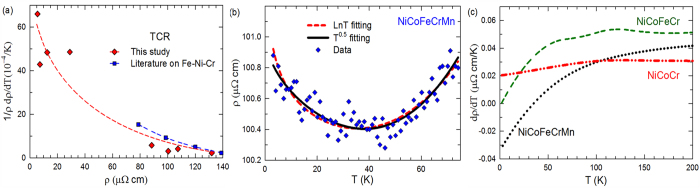
Temperature dependence of electrical resistivity. (**a**) Temperature coefficient of resistivity at 300 K, along with the literature results for various Ni-Fe-Cr alloys. The trend lines are to guide the eyes. (**b**) Kondo-like behavior of NiCoFeCrMn. (**c**) d*ρ*/d*T* of NiCoFeCr, NiCoCr and NiCoFeCrMn.

The temperature coefficient of resistivity (TCR), 1/*ρ dρ*/*dT*, has been considered closely related to the resistivity value^15^,^40^. The TCR usually decreases with increasing resistivity, irrespective of thermal or compositional disorder effects. The thermal contribution is apparent with the temperature dependent expression. The compositional disorder effect on the TCR - ρ correlation in concentrated alloys was reported in the early 1970s by Mooij^15^ for several binary systems such as the Ni-Cr and Ti-Al systems. Later on, experiments on the Ni-Fe-Cr system with various compositions showed a similar trend^40^. The TCR at ~300 K of the 8 measured materials in this study are shown in Fig. 2(a) as a function of resistivity. The literature values of Ni-Fe-Cr are also presented for comparison^40^. The alloys with high resistivities clearly have significantly lower TCR. Both the resistivities and TCR values of these high resistivity alloys are of the same order as those for alloys in the Ni-Fe-Cr system^40^.

Low TCR also appears in other Ni-based concentrated binary alloys. The Ni-Cr system^15^ has a nearly constant resistivity of ~110 μΩ cm at room temperature for Cr concentrations between 20–80%, similar to other high resistivity materials, and the TCRs are similar and on the order of 10^−4^/K. NiCu^16^ has a much lower resistivity of ~40–50 μΩ cm, but its TCR is less than 6 × 10^−4^/K in the temperature range of 100–300 K, which is significantly lower than our trend line. The saturation of resistivity or negative TCR at high temperatures were frequently observed in both amorphous and crystalline high resistivity alloys^15^,^41^, but are not observed in any of our alloys, at least below 300 K. One possible reason for low TCR in high resistivity alloys is related to the Ioffe-Regel limit where the electron mean-free-path approaches the order of the interatomic spacing^15^,^42^.

At lower temperatures, the resistivity due to electron-phonon scattering is not linearly dependent on temperature. The Bloch-Grüneissen formula predicts that the resistivity of simple metals has a *T*^5^ dependence at low temperature^16^. However in the case of transition metals, where electron scattering from *s-* to *d-* bands occurs, this relationship was modified by Wilson *et al.*^43^ into a *T*^3^ dependence, and this relationship has been used successfully to describe the low temperature resistivity of many transition metal alloys^12^,^13^. Thus, in this study, a *T*^3^ rather than a *T*^5^ power law is used to describe the experimental resistivity data.

A resistivity minimum is clearly shown in Fig. 2(b) for the NiCoFeCrMn alloy; it also seems to appear in the NiCoFeCrPd alloy but is much less significant. Such minima in dilute magnetic alloys were first explained by Kondo in 1964^44^, as due to the coupling between the itinerant electrons and localized magnetic impurities. Resonant scattering by magnetic impurities results in a Kondo effect that leads to a –*ln*(*T*) contribution to the resistivity, which when combined with the normal T^3^ or T^5^ contribution results in a resistivity minimum. However, there are other mechanisms that can result in a resistivity minimum. For example in amorphous metals, a −*T*^1/2^ dependence has been observed and explained as a disorder-induced electron-electron interaction^45^. In Ni-Fe-Cr crystalline alloys, experiments in the 1990s also found that the resistivities do not follow –*ln*(*T*), but are better described by −*T*^1/2^ dependence^12^,^18^. We attempted to use both formulas to fit the experimental data for the NiCoFeCrMn alloy and to examine whether one of the formulas provides a better description of the data. As shown in Fig. 2(b), the experimental data are well described by both formulas within the experimental uncertainties; thus, no conclusions regarding these models can be made based on the current results. By including the *T*^2^ factor discussed above, the temperature dependence of resistivity is given by:


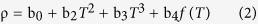


The fit parameters, based on Eq. (2) to the low temperature resistivity data for all 8 alloys are listed in Table 2. The residual resistivities can be approximated as equal to the parameter b_0_. The fourth term, *f* (*T*) stands for either the *ln*(*T*) or *T*^1/2^ dependence for the quinary alloys.

For the NiCoCr alloy, the spline estimation of the derivative of resistivity with temperature, d*ρ*/d*T*, is considerably greater than 0 down to 2 K, as shown in Fig. 2(c). This result suggests the existence of a term linear with *T* in this temperature regime. Actually, the data of NiCoCr can be fitted well by ρ = b_0_ + b_2_*T*^2^ + b_4_*T*. The finite positive d*ρ*/d*T* at such low temperatures is apparently different from the other alloys studied here, as they are either negative (quinary) or 0 (others). A finite positive TCR at low temperatures is rarely observed. There is one report of a finite positive TCR in a Fe_25_Cr_75_ alloy at 0.4 K^12^, but no explanation was given by the authors. Further studies are needed to investigate the origin of this behavior.

### Residual resistivity

In solid solution alloys, residual resistivity arises from the random occupation of underlying lattice sites by the different chemical species comprising the alloy, which then provides a source of electron scattering, and resistivity, even at T = 0 K where the thermal lattice vibrations are absent. Experimentally, comparisons of binary (NiFe vs. NiCo) and ternary (NiCoCr vs. NiCoFe) residual resistivity results indicate that the addition of Cr rapidly increases the resistivity, while the addition of Co has limited effects. Literature results^46^ also show that Ni-Cr binary alloys have a high resistivity of over ~100 μΩ cm, much larger than NiFe and NiCo. Notably, the residual resistivity of the 3-component NiCoCr alloy is larger than that of the 4-component NiFeCoCr, and the 2-component NiFe has larger residual resistivity than the 3-component NiCoFe, while the residual resistivity of 5-component alloys formed by adding either Mn or Pd to NiFeCoCr are the highest. Clearly, the residual resistivity does not have a strong correlation with the number of alloying species. In addition, the difference in their atomic charges contributes only moderately, considering that the alloys containing just Fe, Co and Ni, which order ferromagnetically, have much smaller residual resistivity compared to those that also contain Cr and Mn with complex antiferromagnetic ground states.

### Thermal conductivity

Thermal conductivity (κ) is usually divided into two components, the electronic thermal conductivity (κ_e_) and the lattice thermal conductivity (κ_g_). Direct experimental separation of these two components is not feasible. Thus, to the first order approximation, κ_e_ is usually estimated based on the Wiedemann-Franz relationship


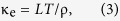


where *L* is the Lorentz constant of 2.45 × 10^−8^ W Ω K^−2 ^47.

**Figure 3:**
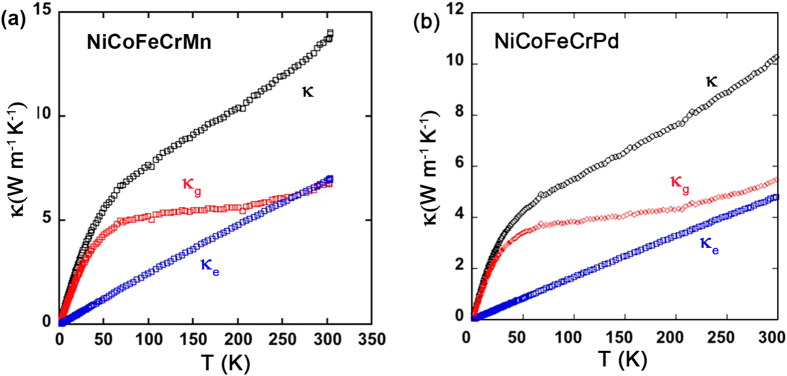
Thermal conductivity of two high entropy alloys. Total, electronic and lattice thermal conductivities of (**a**) NiCoFeCrMn and (**b**) NiCoFeCrPd.

Figure 3 shows the temperature dependence of the measured κ, and the derived values of κ_e_ and κ_g_ for NiCoFeCrMn and NiCoFeCrPd. One distinct feature of the concentrated alloys, relative to pure metals, is that in a pure metal the total thermal conductivity is dominated by κ_e_, and κ_g_ is usually negligible^47^. However, in concentrated alloys, κ_g_ seems to dominate at low temperatures and is comparable with κ_e_ at about room temperature. Chemical disorder appears to scatter electrons more effectively than phonons. In addition, in pure metals κ starts decreasing at a few tens of K, while in concentrated alloys κ keeps increasing up to at least 300 K (see Table 3). One major reason is that, due to the large and nearly constant resistivity of these complex alloys, κ_e_ almost linearly increases with temperature up to 300 K, which is very different from the behavior in pure metals. The theoretical description of the temperature dependence of κ_g_ for disordered alloys has been challenging. However, numerical calculations of κ_g_ in equiatomic binary alloys of NiPd and NiPt^48^ show a very similar temperature dependence to what is shown in Fig. 3: at low temperature, κ_g_ increases rapidly with temperature up to a few tens of K, while its dependence with temperature becomes weaker with increasing temperature, and ultimately becomes nearly constant.

Table 3 lists the κ, κ_e_ and κ_g_ values at 50 and 300 K for the measured alloys. Here we want to emphasize again that the Wiedemann-Franz law is only a coarse approximation; electron-electron interactions, Fermi smearing, and the “vertical” movement in Fermi surfaces may all lead to deviations^47^. Thus for some low-resistivity alloys, application of the Wiedemann-Franz law results in the situation of κ_e_ greater than κ, which is nonrealistic. The thermal conductivity of Ni and NiFe has been previously measured over the temperature regime investigated in this study^49^ and that of NiCoFeCr has been measured at 300 K and above^50^. Our results on these materials agree well with the literature values. The total thermal conductivity of these concentrated alloys are significantly lower than that for pure metals, similar to some super-alloys, but the main reason of the reduction in κ is the low κ_e_ induced by high electrical resistivity. Recent atomic simulations^11^ using Lennard-Jones potentials suggest that scattering of phonons can be tailored by varying the composition of alloys with multiple principal elements. However, it is hard to verify this statement based on the present experimental results, as the derived κ_g_ values do not show a clear relationship with composition and the number of elements beyond the potential error induced from the use of the Wiedemann-Franz law.

### Magnetic properties

The magnetic states of high entropy alloys have been of interest recently. The Curie temperature *T*_c_, and the saturation magnetization *M*_s_ have been identified in NiCoFe, NiCoFeCr and NiCoFeCrPd^9^,^13^,^19^,^20^. For these three materials, our measurements show similar results: the *M*_*s*_ at 5 K for these three materials are 1.7, 0.24, and 0.52 μ_B_/atom, respectively. The *T*_c_ of NiCoFeCr is ~120 K, while the other two materials remain ferromagnetic at room temperature. For the saturation magnetization, the experimental results are in reasonable agreement with our calculated values of the configurationally averaged local moments, 1.6, 0.66, and 0.63 μ_B_/atom, using the KKR-CPA. The larger discrepancy between the experiment and theory for NiCoFeCr may well be indicative of a more complex, non-collinear, ground state than allowed by the KKR-CPA calculations, which was restricted to collinearity.

**Figure 4:**
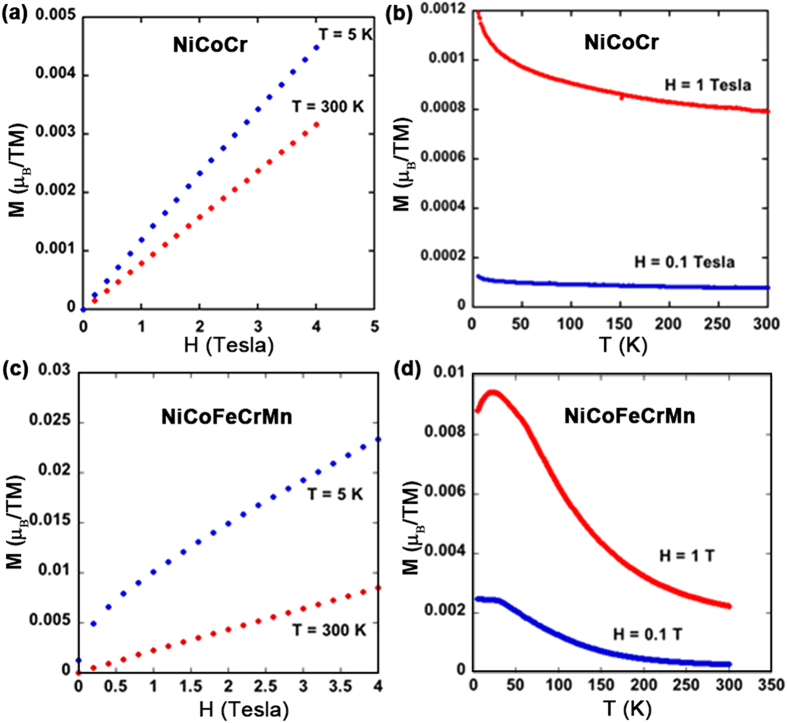
Magnetic properties of NiCoCr and NiCoFeCrMn. Magnetization and temperature dependence of (**a**,**b**) NiCoCr and (**c**,**d**) NiCoFeCrMn, from 4 to 300 K.

Here we further present magnetization data for the other two materials, NiCoCr (shown in Fig. 4a,b) and NiCoFeCrMn (shown in Fig. 4c,d). NiCoCr is paramagnetic above 4 K. The magnetic susceptibilities at 5 K and 300 K are ~1.1 × 10^−5^ and 8 × 10^−6^ emu/g, respectively. The decrease of *T*_c_ from NiCoFe, NiCoFeCr to NiCoCr shows that the addition of Cr significantly reduces the magnetization^19^. This is attributed to magnetic cancellation because the magnetic moment of Cr is anti-parallel to that of the other elements in the alloys as is also found in our KKR-CPA studies. The *M*-*T* curve of NiCoFeCrMn at high magnetic field (1 T) exhibits a peak at ~25 K. This suggests either a type of antiferromagnetic ordering or possibly a spin glass transition. Further magnetization, ac susceptibility, heat capacity and possibly neutron scattering experiments are needed to conclusively determine the origin of the peak in the susceptibility near 25 K. At the transition temperature for both NiCoFeCr and NiCoFeCrMn, we note that no significant change in the temperature dependence of electrical resistivity is observed, probably because the contribution of spin-disorder scattering is small, as compared with the scattering from electrons and phonons at these temperatures.

## Discussions

**Figure 5:**
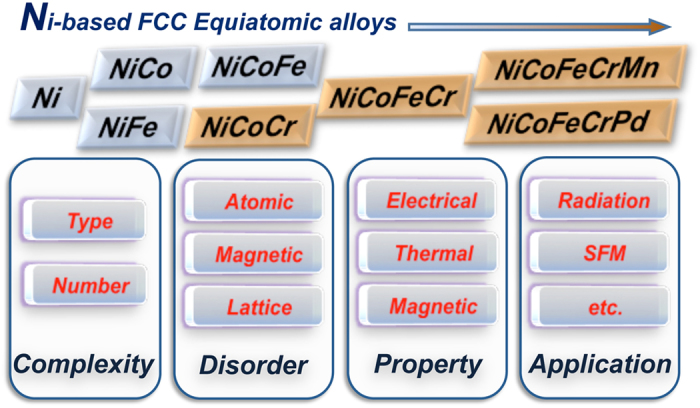
Effects of chemical complexity on the physical properties by introducing different types of disorder. Type and number of alloying elements are two important factors controlling the chemical complexity in equiatomic alloys. Ni, NiCo, NiFe and NiCoFe (without addition of Cr) have smaller disorder and thus lower electron scattering; NiCoCr, NiCoFeCr, NiCoFeCrMn and NiCoFeCrPd have relatively larger disorder and thus higher electron scattering. Such effects on the physical properties will further influence their practical applications.

Our findings systematically show that, although the physical properties (especially for ρ and κ_e_) in this study have some correlations with the number of elements, e.g. the quinary alloys have the highest electrical resistivity and the lowest thermal conductivity, they more strongly depend on the type of alloying elements, e.g. the addition of Cr. The analysis, thus, must go down to the disorder level to understand such correlations. While the electron scattering (at 0 K) in disordered alloys is ultimately due to compositional disorder, it is useful to identify several different contributions: e.g. atomic, magnetic and displacement-disorder (see Fig. 5). The former two refer to the intrinsic disorder associated with the particular alloying species on the sites of an underlying ideal crystalline lattice (e.g. FCC) and the extent that some species may generate a magnetic moment. The latter refers to the additional displacement disorder associated with local structural distortions that result from the different chemical environments surrounding each atom.

**Figure 6:**
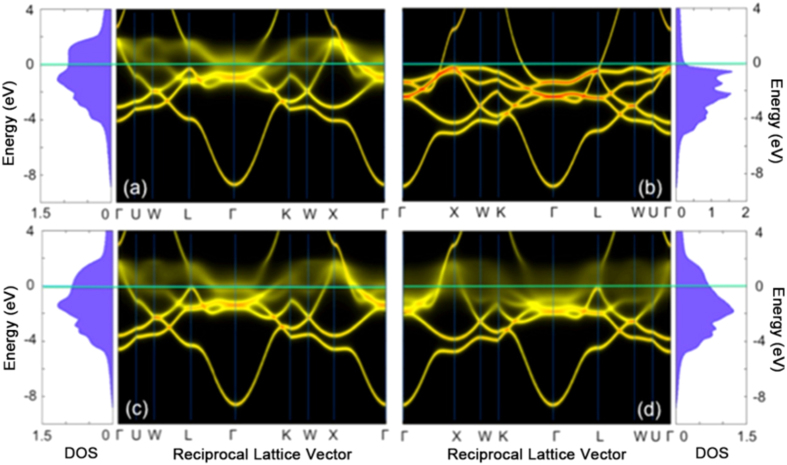
Comparison of electronic structures between NiCoFe and NiCoCr by KKR-CPA calculations. Calculated KKR-CPA BSF and DOS for equiatomic FCC NiCoFe (**a**,**b**) and NiCoCr (**c**,**d**) disordered solid solution alloys for the majority (**b**,**d**) and minority (**a**,**c**) spin channels respectively.

To shed light on the connections between the complexity/disorder and the experimental electrical properties, in Fig. 6, we show the results of calculations based on the *ab initio* KKR-CPA method of the electronic structure of exemplary low (NiCoFe) and high (NiCoCr) RR alloys (see Fig. 1). Figure 6 shows results for the configurationally averaged density of states (DOS) and Bloch spectral function (BSF) for the two alloys resolved in majority (b, d) and minority (a, c) spin components. For the NiCoFe majority spin channel, the DOS and BSF both exhibit sharp structure, very similar to that of a pure metal, while for the minority channel the DOS are broadened in both energy and wave-vector. The Fermi energy wave vector broadening of the BSF is related to the inverse of the electron mean-free-path. While this is short for the minority spin electrons, it is large for the majority spin electrons thereby providing a short circuit and an overall low resistivity. On the contrary, for NiCoCr both channels are broadened particularly in the vicinity of the Fermi energy implying a short mean free path in both spin channels and correspondingly high RR. The underlying reason for contrasting behavior is related to the differences in the magnetic interactions among the alloy species. For the NiCoFe alloys we find a simple ferromagnetic solution where the local moments of Ni, Co and Fe all point in the same direction (e.g. “up”). In NiCoCr we find that, while the Ni and Co local moments point up, the moments on the Cr site are antiparallel to them, or “down”. Thus a spin-up electron leaving an up moment site (say a Ni-site) will encounter scattering potential in which the “up” and “down” scattering potentials have been reversed when encountering a down moment Cr-site, resulting in strong scattering; the same argument in reverse applies to the down spin electron resulting in strong scattering in both channels.The low residual resistivity situation found in NiCoFe is very similar to that found in NiFe. Here for the high RR alloys, we can attribute this to the mixed ferro/antiferro-magnetic state that provides an additional source of disorder scattering in alloys containing a mixture of ferromagnetically coupled (Ni, Co, Fe) and antiferromagnetically coupled (Cr and Mn) elements.

While the KKR-CPA calculations of the DOS and BSF provide a basis for understanding the large variations in RR found in the alloys studied here, a detailed understanding will require a full calculation of the residual resistivity within the KKR-CPA^24^,^51^,^52^ to delineate the extent to which the residual resistivity can be accounted for by potential scattering induced by atomic and magnetic disorder alone. Because the KKR-CPA is an effective medium theory that, on average, restores the underlying (in this case FCC) lattice periodicity, any effects of displacement fluctuations are neglected. Clearly, the differences between the measured residual resistivity and those based on KKR-CPA will allow attention to be focused on the additional role of displacement fluctuations. That the scattering of electrons of NiCoFeCrPd is largest of all of the alloys studied is most likely due to the increased importance of displacement fluctuation scattering when the larger *4d* Pd-atom is alloyed with the other *3d*-elements. Despite these remaining issues to be further explored, the KKR-CPA calculations have well explained, from the perspectives of chemical and magnetic disorders, the large separation between the alloys with low resistivity and those with high resistivity, and explained why certain alloying species, rather than the number of alloying elements have more critical impact on the transport properties.

## Conclusions

Electrical resistivities of Ni and seven Ni-based FCC equiatomic alloys have been measured from 4 to 300 K. While the quinary alloys have the highest resistivities, the type of the elements has a larger influence than the number of elements. The addition of Cr significantly enhances the scattering of electrons in Ni-based FCC equiatomic alloys. The high resistivity alloys have low TCR values. The two quinary alloys show a Kondo-like behavior at tens of K, and NiCoCr has a finite positive TCR at very low temperature. The *ab* initio KKR-CPA calculations reveal that the high residual resistivity of the alloys containing Cr and Mn is strongly attributed to the magnetic disorder.

Thermal conductivity of the alloys is significantly lower than that of pure metals, primarily due to the high electrical resistivity and the resulting suppression of the electronic thermal conductivity. The temperature dependence of the derived lattice thermal conductivities of the two quinary alloys show two behaviors: below ~50 K, the lattice thermal conductivity increases with increasing temperature, while at higher temperatures it seems to saturate.

The magnetic properties of these alloys have been measured in magnetic fields up to 4 T and in the temperature range of 4–300 K. NiCoCr is paramagnetic in this range, while NiCoFeCrMn might have a spin glass transition at ~25 K. The magnetic measurements on the other alloys are consistent with recent literature results.

## Methods

### Materials preparation

The alloy ingots were prepared by first arc-melting together appropriate amounts of the pure Ni, Co, Fe, Cr, Mn and Pd elemental metals (>99.9% pure). To ensure a homogeneous well-mixed alloy, each composition (arc-melted button) was flipped and re-melted five times before drop-casting the melt into a copper mold. The polycrystalline drop-cast ingots (~10–12 mm in diameter) were then loaded into an optical floating zone furnace and converted to a single crystal. The details of the material preparation and crystal growth can be found elsewhere^34^,^53^. X-ray diffraction and metallographic examination were used to check the crystal/microstructure of the as-grown rods. Ni, NiCo, NiFe, NiCoFe, NiCoCr and NiCoFeCr were confirmed to be single crystals. NiCoFeCrMn and NiCoFeCrPd were polycrystalline with mm-size grains. These grains are much larger than either electron or phonon mean-free-paths; hence, the associated grain boundaries have no measurable effect on either electrical or thermal transport values. All the samples were cut into bars with dimensions of ~1 × 1 × 10 mm^3^ using electro-discharge machining (EDM), and the bars were electrolytically polished to remove the EDM-damaged layers before measurements.

### Physical property measurements

Electrical and thermal transport measurements were performed in a Quantum Design Physical Property measurement system using both the Resistivity and Thermal Transport Options. Silver epoxy (H20E Epo-Tek) was used for electrical, thermal, and mechanical contacts in a standard four-point configuration. For temperature (4–300 K) and magnetic field dependent (0–4 Tesla) magnetization measurements, a Quantum Design SQUID magnetometer was used.

### KKR-CPA calculation

*Ab* initio KKR-CPA calculations were performed using the Hutsepot code. Implemented with density functional theory (DFT) the KKR-CPA provides a first-principles description of the effects of the configurationally averaged properties within a mean field, or single-site approximation^14^. In this study, the calculations were performed based on the ideal FCC structure with experimentally determined lattice parameters for each alloy. Local spin density approximation (LDA) was used for the exchange correlation, and atomic sphere approximation (ASA) was applied for the crystal potential. The KKR-CPA calculations were performed spin-polarized to allow for the possibility of a magnetic ground state. Collinear ground state solutions were obtained in all of the alloys studied in this paper. An angular momentum cut-off of 3 was used in the solution of the multiple-scattering equations. The KKR-CPA scattering-path matrix was calculated in reciprocal (k) space using a 40 × 40 × 40 k-point mesh during the DFT self-consistency cycle and 50 × 50 × 50 k-point mesh for the density of states (DOS) calculation. The DOS and Bloch spectral function (BSF) were calculated for an energy mesh spacing of 0.001 Ry along the real energy axis and at an energy of 0.001 Ry off into the upper half of the complex plane. The BSF were calculated for a dense k-point mesh along the high symmetry directions, for example a total of 200 equally spaced k-points were used along Γ-X in the FCC Brilloin zone.

## Additional Information

**How to cite this article**: Jin, K. *et al.* Tailoring the physical properties of Ni-based single-phase equiatomic alloys by modifying the chemical complexity. *Sci. Rep.* **6**, 20159; doi: 10.1038/srep20159 (2016).

## Figures and Tables

**Table 1:** Temperature dependence of electrical resistivity at high temperature range, fit by ρ = a_0_ + a_1_*T* + a_2_*T*^2^.

	Range (K)	a_0_ (μΩ cm)	a_1_ (10^−2^ μΩ cm K^−1^)	a_2_ (10^−4^ μΩ cm K^−^)	ρ_300K_ (μΩ cm)
Ni	>100	−0.97	1.56	0.43	7.5
NiCo	>100	1.30	1.68	0.29	9
NiFe	>100	9.26	1.72	2.48	37
NiCoFe	>230	1.66	2.65	0.70	16
NiCoCr	>100	92.74	3.07	–	102
NiCoFeCr	>100	75.70	5.06	–	91
NiCoFeCrMn	>150	97.65	3.43	0.19	110
NiCoFeCrPd	>100	124.78	2.86	–	134

**Table 2:** Temperature dependence of electrical resistivity at low temperature range, fit by ρ = b_0_ + b_2_*T *^2^ + b_3_*T *^3^ + b_4_*f*(*T*).

	Range (K)	b_0_ (μΩ cm)	b_2_ (10^−4^ μΩ cm K^−2^)	b_3_ (10^−6^ μΩ cm K^−3^)	b_4_*f*(*T*) (10^−2^)
Ni	<75	0.02	0.16	1.12	
NiCo	<75	2.07	0.29	1.23	/
NiFe	<75	10.37	1.54	1.90	/
NiCoFe	<80	4.87	0.70	0.62	/
NiCoCr	<75	93.21	0.52	/	2.04 (*T*)
NiCoFeCr	<30	77.15	1.78	10.7	/
NiCoFeCrMn	<75	101.04	0.19	1.96	−12.4 (*T*^0.5^)
		101.20	0.19	1.48	−25.0 (Ln *T*)
NiCoFeCrPd	<75	126.63	1.64	0.10	−7.18 (*T*^0.5^)
		126.66	1.20	0.33	−11.4 (Ln *T*)

**Table 3:** Total thermal conductivity (κ) and an estimate of the electronic (κ_e_) and lattice contributions (κ_g_) for the eight materials at 50 and 300 K.

	κ	κ_e_ 50 K	κ_g_	κ	κ_e_ 300 K	Κ_g_
Ni	328	651	–	88	88	–
NiCo	51.4	48.1	3.3	69.9	74	–
NiFe	15.4	11.4	4	28.0	21.1	6.9
NiCoFe	26.9	23.1	3.8	43.3	46.8	–
NiCoCr	8.3	1.3	7.0	11.4	7.2	4.2
NiCoFeCr	6.2	1.5	4.7	12.8	8.0	4.8
NiCoFeCrMn	5.6	1.3	4.3	13.7	6.8	6.9
NiCoFeCrPd	4.2	0.8	3.4	10.3	4.8	5.5

The values are in the unit of W m^−1^ K^−1^.
